# Prevalence and associated factors of diabetic kidney disease among rural patients with type 2 diabetes mellitus in Guangxi, China: a cross-sectional study

**DOI:** 10.3389/fcdhc.2026.1706901

**Published:** 2026-02-27

**Authors:** Yelan Huang, Jingfeng Chen, Chaoqun Bai, Xiaoxue Lei, Yanping Zhang, Guifen Fu

**Affiliations:** 1 School of Nursing, Guangxi University of Chinese Medicine, Nanning, China; 2 Department of Geriatric Endocrinology and Metabolism, Guangxi Academy of Medical Sciences and the People’s Hospital of Guangxi Zhuang Autonomous Region, Nanning, China; 3 Department of Nursing, Guangxi Academy of Medical Sciences and the People’s Hospital of Guangxi Zhuang Autonomous Region, Nanning, China

**Keywords:** associated factors, cross-sectional study, diabetic kidney disease, prevalence, rural health, type 2 diabetes mellitus

## Abstract

**Objective:**

To investigate the prevalence of diabetic kidney disease (DKD) and identify its associated factors among rural patients with type 2 diabetes mellitus (T2DM) in Guangxi, China.

**Methods:**

A multistage stratified random sampling method was applied. Five cities across Guangxi (Nanning, Guilin, Hechi, Chongzuo, and Yulin) were randomly selected, followed by three counties in each city, yielding 15 counties in total. One hospital from each county was chosen, and patients with T2DM were recruited for questionnaires and laboratory testing. Descriptive epidemiological methods were used to calculate the prevalence of DKD. Univariate and multivariate logistic regression analyses were performed to identify associated factors.

**Results:**

A total of 2,178 rural patients with T2DM were included (1,204 men and 974 women; mean age 63.25 ± 12.71 years; mean disease duration 7.96 ± 4.07 years). The HbA1c control rate was 22.68%, and the prevalence of DKD was 23.32% (95% CI: 21.6% to 25.1%; 508/2,178). Logistic regression analysis showed that higher diabetes knowledge levels were protective against DKD (OR = 0.936, 95% CI: 0.891–0.984). Independent associated factors. included elevated HbA1c (OR = 1.530, 95% CI: 1.389–1.686), serum creatinine (OR = 1.037, 95% CI: 1.033–1.041), uric acid (OR = 1.005, 95% CI: 1.004–1.007), and triglycerides (OR = 1.190, 95% CI: 1.010–1.403).

**Conclusion:**

The prevalence of DKD among rural patients with T2DM in Guangxi is relatively high. Enhancing diabetes-related knowledge and strengthening the monitoring and control of HbA1c, lipid profiles, creatinine, and uric acid may help reduce the risk of DKD and improve patient outcomes.

## 1 Introduction

Diabetes mellitus (DM) is a chronic metabolic disorder characterized by persistent hyperglycemia, resulting from defects in insulin secretion, insulin action, or both. Prolonged hyperglycemia contributes not only to macrovascular complications such as coronary heart disease and stroke, but also to microvascular complications including diabetic kidney disease (DKD) and diabetic retinopathy (1).

According to the *International Diabetes Federation (IDF) Diabetes Atlas, 10th edition*, 537 million people worldwide were living with diabetes in 2021, and this number is projected to rise to 643 million by 2030 and 783 million by 2045. In addition, an estimated 541 million adults had impaired glucose tolerance in 2021 (2). In China, the prevalence of diabetes has reached 11.6%, with type 2 diabetes mellitus (T2DM) accounting for more than 90% of cases (3). The mortality rate among rural diabetic patients in China is increasing rapidly (4). This trend poses a major challenge to healthcare systems due to the dual burden of rising disease prevalence and associated healthcare costs (5). An investigation conducted among the Zhuang ethnic population in Nanning, Guangxi, revealed an overall prevalence of abnormal glucose metabolism of 37.52%, with Type 2 diabetes mellitus (T2DM) and prediabetes (PDM) accounting for 9.7% and 23.17% of cases, respectively (6). Persistent hyperglycemia leads to widespread endocrine and metabolic disturbances, which in turn contribute to the development of multiple diabetes-related complications (7). Poor glycemic control significantly elevates morbidity and mortality, including risks of stroke, renal insufficiency, lower-limb amputation, cardiovascular disease, vision loss, and neuropathy.

Among microvascular complications, DKD is one of the most severe. It has become the leading cause of chronic kidney disease (CKD) and end-stage renal disease (ESRD) in both China and worldwide (8). Epidemiological studies suggest that 30%–40% of patients with diabetes eventually develop DKD (9, 10). With the global rise in diabetes prevalence, the incidence of DKD continues to increase, placing a heavy burden on patients’ quality of life and on healthcare systems.

Although treatment options for DKD have advanced in recent years, early diagnosis and effective prevention remain limited. The disease involves complex pathophysiological mechanisms, making intervention challenging. Thus, identifying associated factors and implementing timely interventions are crucial for delaying disease progression. This study investigated the prevalence and risk factors of DKD among rural patients with diabetes in Guangxi, China, with the aim of providing evidence to guide prevention and management strategies for DKD in this population.

## 2 Methods

### 2.1 Study population

A pilot survey was conducted among 150 rural patients with T2DM from 15 counties (10 patients per county), of whom 116 had diabetes-related complications, yielding a prevalence of 77.33%. Using the multistage stratified sampling formula (11), n = Z^2^_1-α/2_(1-P)P/δ^2^×deff, with *Z*_1−_α/2 = 1.96, *P* = 77.33% (prevalence from the pilot study), *deff* = 2 (design effect to account for regional differences), and δ = 0.1 (tolerable error), the required sample size per county was calculated to be 136. To account for potential data loss, the sample size was increased by 10%, resulting in a final target of 152 patients per county. Accordingly, a total of 2,280 participants from 15 counties were planned for enrollment.

The inclusion criteria were as follows: (1) diagnosis of T2DM according to the 2020 Chinese Diabetes Society criteria (12); (2) age ≥ 18 years; and (3) permanent household registration and residence in the study area.

Diagnosis of Diabetic Kidney Disease (DKD): For the purpose of this study, DKD was defined based on clinical criteria as recommended by the Chinese Diabetes Society (12). Specifically, DKD was diagnosed by the presence of persistent albuminuria (urinary albumin-to-creatinine ratio ≥ 30 mg/g) and/or reduced estimated glomerular filtration rate (eGFR < 60 mL/min/1.73 m²) in patients with diabetes.

The exclusion criteria were: (1) patients with gestational diabetes. (2) patients with dementia or psychiatric disorders, based on medical records or family reports. (3) Patients with known or suspected non-diabetic kidney diseases, including but not limited to hypertensive nephropathy, chronic glomerulonephritis, polycystic kidney disease, obstructive nephropathy, or other primary renal diseases. (This exclusion was based on medical record review, patient self-report of a prior diagnosis, or clinical presentation suggestive of a non-diabetic etiology). (4) Patients with acute kidney injury. (5) Patients with an active urinary tract infection. (6) Pregnant patients. (7) Patients in the acute phase of any other illness who were unable to participate.

Institutional approval was obtained from all participating hospitals, and written informed consent was provided by all participants.

### 2.2 Questionnaire survey

A self-designed general information questionnaire was used to collect demographic characteristics (age, sex, marital status, education level, annual per capita household income, smoking status, and drinking status) as well as disease-related information (duration of diabetes, HbA1c level, use of oral hypoglycemic agents or insulin, presence of complications, and family history of diabetes).

Diabetes Knowledge Scale: This instrument was originally developed by Speight and Bradley at the University of London in 2001 (13) and revised in 2003. It consists of 26 dimensions and 110 items covering treatment, dietary management, physical activity, hypoglycemia management, foot care, the consequences of smoking and drinking, sick-day management, and complication prevention. Each item had three response options: correct, incorrect, or “don’t know”. Correct responses were scored as 1, with higher total scores indicating greater diabetes-related knowledge. The Cronbach’s α coefficient for the Chinese version was 0.909, indicating good internal consistency reliability.

Social Support Rating Scale (SSRS): Developed by Xiao (14), this instrument consists of 12 items across three dimensions: subjective support, objective support, and utilization of support. Total scores range from 12 to 66, with higher scores indicating greater levels of social support. In the present study, the Cronbach’s α coefficient was 0.838, demonstrating acceptable reliability.

#### 2.2.1 Laboratory measurements

After an overnight fast of at least 10 hours, venous blood samples were collected from all participants. Serum was separated by centrifugation and used for the analysis of lipid profiles, creatinine, and uric acid. A separate whole blood sample collected in an EDTA tube was used for the HbA1c analysis.

All laboratory analyses were performed in the central laboratories of the participating hospitals using standardized automated clinical analyzers. The specific methods were as follows:

1. HbA1c was measured by high-performance liquid chromatography (HPLC).
2. Lipid profiles (including triglycerides) were measured using enzymatic methods.
3. Serum creatinine was measured using the creatinine oxidase method.
4. Uric acid was measured using the uricase-peroxidase method.

All laboratory procedures followed the manufacturers’ instructions and adhered to strict quality control standards.

### 2.3 Definitions

1. Glycemic control: HbA1c < 7.0% was defined as controlled glycemia, according to the Chinese Guidelines for the Prevention and Treatment of Type 2 Diabetes (2020 edition) (12). The control rate (%) was calculated as:Control rate=Number of controlled patients Total/surveyed patients×100%
2. Smoking status: Smoking was defined as continuous or cumulative smoking for ≥ 6 months. Based on their current smoking frequency, participants were categorized as: regular smokers (smoking ≥ 1 cigarette per day on average), occasional smokers (smoking at least once per week but less than 1 cigarette per day on average), or former smokers (having met the smoking definition but having quit for > 6 months). For the purpose of logistic regression analysis, a binary variable was created by combining regular and occasional smokers as “smokers” and combining former smokers with those who never smoked as “non-smokers”.
3. Alcohol consumption: Drinking was defined as consuming any alcoholic beverage at least once per month for ≥ 6 months. Based on their current drinking frequency, participants were categorized as: regular drinkers (drinking ≥ 1 time per week), occasional drinkers (drinking at least once per month but less than once per week), or former drinkers (having met the drinking definition but having quit for > 6 months). For the purpose of logistic regression analysis, a binary variable was created by combining regular and occasional drinkers as “drinkers” and combining former drinkers with those who never drank as “non-drinkers”.

### 2.4 Ethical considerations

This study was approved by the Ethics Committee of the authors’ institution (Approval No: KT-KJT-2021-26). All questionnaires emphasized that participation was voluntary and confidential. Written informed consent was obtained from all participants as well as from the administrators of the participating hospitals.

### 2.5 Data collection

The study involved questionnaire surveys and standardized venous blood collection. Demographic and clinical information, as well as laboratory measurements including HbA1c, lipid profiles, creatinine, and uric acid, were collected from 15 county hospitals. Data were entered into Excel spreadsheets and uploaded via the internet. Two researchers independently verified the datasets before importing them into SPSS for statistical analysis. All electronic files were stored on secure, password-protected computers to ensure confidentiality.

### 2.6 Statistical analysis

The final analytical sample comprised 2,178 patients (95.6% of the initially enrolled 2,280). Missing data included 35 cases with missing laboratory/examination results and 67 cases with missing or incomplete questionnaire data, totaling 102 cases (4.5%). A complete-case analysis approach was employed for data handling. Data were analyzed using SPSS version 25.0. Continuous variables with a normal distribution were presented as mean ± standard deviation (SD) and compared using independent-samples *t* tests. Categorical variables were presented as frequencies and compared using χ² tests. Multivariate logistic regression analysis was performed to identify associated factors for DKD. A two-tailed *p* value < 0.05 was considered statistically significant.

## 3 Results

### 3.1 Baseline characteristics of participants

A total of 2,280 rural patients with T2DM were enrolled in the survey, and 2,178 valid questionnaires were obtained. The mean age of participants was 63.25 ± 12.71 years, and the mean duration of diabetes was 7.96 ± 4.07 years. Of the study population, 1,204 (55.28%) were male and 974 (44.72%) were female. The detailed demographic and clinical characteristics of the participants are summarized in **Table 1**.

**Table 1:** General information of surveyed rural diabetic patients (n=2178).

Variable	Group	Count (Percentage, %)
Sex	Male	1204 (55.28)
	Female	974 (44.72)
Marital Status	Married	2020 (92.75)
	Unmarried or Widowed	158 (7.25)
Educational Level	Illiterate	256 (11.75)
	Primary school	504 (23.14)
	Middle school	684 (31.41)
	High school	507 (23.28)
	College diploma	180 (8.26)
	Bachelor’s degree	47 (2.16)
Average Family Disposable Income	<18,931 yuan	1356 (62.26)
	≧18,931 yuan	822 (37.74)
Family History of Diabetes	Yes	317 (14.55)
	No	1861 (85.45)
HbA1c Control Status	< 7.0	494 (22.68)
	≥ 7.0	1684 (77.32)

### 3.2 Prevalence of DKD in rural patients with diabetes

The prevalence of DKD among rural patients with T2DM was 23.32% (95% CI: 21.6% to 25.1%; 508/2,178). Comparative analysis revealed significant differences between patients with and without DKD in sex, educational level, household income, disease duration, family history of diabetes, use of oral hypoglycemic agents, insulin therapy, HbA1c, creatinine, uric acid, triglycerides, high-density lipoprotein (HDL), diabetes knowledge scores, and social support scores (*p* < 0.05 for all comparisons). Detailed results are presented in **Table 2**.

**Table 2:** Baseline characteristics of rural diabetes patients with and without DKD (n = 2,178).

Variable	Total (n)	With DKD (n = 508)	Without DKD (n = 1,670)	Test statistic	p-value
Age (years)	2,178	63.14 ± 12.92	63.29 ± 12.65	t = 2.221	0.825
Sex				χ² = 17.608	<0.001
Male	1,204	322	788		
Female	974	186	882		
Education level				χ² = 31.473	<0.001
Illiterate	256	84	172		
Primary school	504	134	367		
Middle school	684	154	530		
High school	507	92	415		
College diploma	180	37	143		
Bachelor’s degree	47	4	43		
Marital status				χ² = 0.001	0.997
Married	2,020	471	1,549		
Unmarried or Widowed	158	37	121		
Average Family Disposable Income				χ² = 22.843	<0.001
<18,931 yuan	1,356	362	994		
≧18,931 yuan	822	146	676		
Smoking				χ² = 0.329	0.566
Yes	396	88	308		
No	1,782	420	1,362		
Alcohol consumption				χ² = 0.160	0.689
Yes	496	119	377		
No	1,682	389	1,293		
Duration of diabetes (years)				χ² = 24.168	<0.001
0 - 5	465	74	391		
5 - 10	1,134	267	867		
≧ 10	579	167	412		
Family history of diabetes				χ² = 4.010	0.045
Yes	317	60	257		
No	1,861	448	1,413		
Use of oral hypoglycemic agents				χ² = 20.742	<0.001
Yes	1,349	271	1,078		
No	829	237	592		
Insulin therapy				χ² = 45.862	<0.001
Yes	913	147	766		
No	1,265	361	904		
HbA1c (%)		9.94 ± 1.94	8.20 ± 2.09	t = 16.763	<0.001
Creatinine (µmol/L)		175.65 ± 63.51	87.65 ± 41.37	t = 36.512	<0.001
Uric acid (µmol/L)		369.19 ± 102.90	285.41 ± 105.27	t = 15.789	<0.001
Total cholesterol (mmol/L)		5.27 ± 2.48	5.27 ± 2.36	t = 0.020	0.984
Triglycerides (mmol/L)		2.37 ± 0.87	2.21 ± 1.01	t = 3.268	0.001
HDL (mmol/L)		1.21 ± 0.34	1.17 ± 0.36	t = 2.287	0.022
LDL (mmol/L)		2.93 ± 0.94	2.85 ± 1.07	t = 1.581	0.114
Diabetes knowledge score		44.56 ± 4.10	47.70 ± 4.65	t = 13.699	<0.001
Social support score		23.54 ± 7.45	27.66 ± 10.38	t = 8.329	<0.001

Data are presented as mean ± standard deviation for continuous variables and number (percentage) for categorical variables.

### 3.3 Logistic regression analysis of associated factors for DKD

Multivariate logistic regression analysis was performed with DKD status as the dependent variable and demographic, clinical, and laboratory factors as independent variables. The results indicated that higher diabetes knowledge scores were protective against DKD (OR = 0.936, 95% CI: 0.891–0.984, *p* = 0.010). Independent associated factors included elevated HbA1c (OR = 1.530, 95% CI: 1.389–1.686, *p* < 0.001), elevated creatinine (OR = 1.037, 95% CI: 1.033–1.041, *p* < 0.001), elevated uric acid (OR = 1.005, 95% CI: 1.004–1.007, *p* < 0.001), and elevated triglycerides (OR = 1.190, 95% CI: 1.010–1.403, *p* = 0.038). The detailed results of the regression analysis are summarized in **Table 3**.

**Table 3:** Logistic regression analysis of factors associated with diabetic kidney disease among rural patients with diabetes.

Variable	β	SE	Wald χ²	p-value	OR (95% CI)
Constant	-9.923	1.674	35.126	–	–
Sex					
Female	–	–	–	–	–
Male	0.016	0.169	0.009	0.925	1.016 (0.730, 1.414)
Education level			4.340		
Illiterate	–	–	–	–	–
Primary school	0.710	0.716	0.982	0.322	2.034 (0.499, 8.281)
Middle school	0.939	0.692	1.840	0.175	2.556 (0.659, 9.923)
High school	0.933	0.685	1.852	0.174	2.541 (0.663, 9.738)
College diploma	0.760	0.685	1.229	0.268	2.138 (0.558, 8.193)
Bachelor’s degree	1.191	0.725	2.696	0.101	3.291 (0.794, 13.641)
Average Family Disposable Income					
<18,931 yuan	–	–	–	–	–
≧18,931 yuan	-0.034	0.177	0.036	0.849	0.967 (0.683, 1.368)
Duration of diabetes (years)					
0 - 5	–	–	–	–	–
5 - 10	0.393	0.212	3.436	0.064	1.481 (0.978, 2.244)
≧ 10	0.364	0.241	3.278	0.131	1.438 (0.897, 2.306)
Family history of diabetes					
No	–	–	–	–	–
Yes	0.182	0.239	0.579	0.447	1.200 (0.751, 1.917)
Use of oral hypoglycemic agents					
No	–	–	–	–	–
Yes	0.046	0.159	0.084	0.772	1.047 (0.766, 1.431)
Insulin therapy					
No	–	–	–	–	–
Yes	0.010	0.174	0.003	0.956	1.010 (0.718, 1.419)
HbA1c (%)	0.425	0.049	74.036	<0.001	1.530 (1.389, 1.686)
Uric acid (µmol/L)	0.005	0.001	55.834	<0.001	1.005 (1.004, 1.007)
Creatinine (µmol/L)	0.036	0.002	398.952	<0.001	1.037 (1.033, 1.041)
Triglycerides (mmol/L)	0.174	0.084	4.311	0.038	1.190 (1.010, 1.403)
HDL (mmol/L)	-0.503	0.219	0.415	0.519	0.623 (0.450, 0.975)
Social support score	-0.014	0.010	1.888	0.169	0.986 (0.967, 1.006)
Diabetes knowledge score	-0.066	0.025	6.665	0.010	0.936 (0.891, 0.984)

Variables marked with “–” in the p-value column serve as the reference category for that variable in the regression model.

## 4 Discussion

Diabetes is typically characterized by chronic hyperglycemia and progressive vascular injury. Diabetic complications are major threats to the health and survival of patients with diabetes. Persistent hyperglycemia is the primary clinical feature of diabetes mellitus (DM); the resulting endocrine and metabolic disturbances can cause long-term damage to multiple organs and tissues, leading to a range of diabetes-related complications (15). Diabetic kidney disease (DKD), also referred to as diabetic nephropathy (DN), is one of the most severe microvascular complications of DM. As DKD advances, it may ultimately lead to renal failure and thereby seriously compromise patient health and survival (16). To reduce the burden of microvascular complications in patients with diabetes, this study focused on identifying the relevant associated factors to inform clinical prevention and management strategies.

In this study, we investigated 2,178 rural patients with diabetes in Guangxi and found that the prevalence of DKD was as high as 23.32% (508/2,178). This rate is higher than that reported in previous studies (17). Two main factors may explain this result. First, rural residents in Guangxi often have fixed dietary patterns, limited physical activity, and generally lower levels of health-related behaviors, which substantially increase the likelihood of developing diabetic kidney disease. Second, the study population consisted entirely of patients who actively sought medical care in county hospitals. Some of these patients may have presented with symptoms of complications, which likely contributed to the relatively high prevalence of DKD in the study sample.

To further explore the factors associated with DKD among rural patients with diabetes in Guangxi, this study collected detailed clinical data and laboratory indicators. Our results showed that higher levels of diabetes-related knowledge were protective against diabetic kidney disease. Improved knowledge helps patients understand the importance of glycemic control, including diet management, medication adherence, physical activity, and blood glucose monitoring (18). Effective glycemic control can delay the progression of diabetic kidney disease (19). Patients with greater knowledge are also more likely to participate actively in disease management, such as attending regular follow-up visits and undergoing microalbuminuria testing (20, 21). Therefore, to translate this knowledge-protective effect into clinical practice, we propose that future health initiatives in this region adopt culturally-adapted and practical strategies. These should include establishing village-level, peer-led education programs delivered in the local primary language or dialect (e.g., Zhuang) to maximize comprehension and engagement, focusing on self-management skills and awareness of renal complications. This should be coupled with the use of accessible mobile health technology (e.g., automated text or voice reminders in the appropriate local language) to improve adherence to medication and regular screening protocols for microalbuminuria.

However, an alternative explanation must be considered due to the cross-sectional nature of our study. Patients who have already developed early signs of kidney complications (e.g., detected proteinuria) or have been diagnosed with DKD may become more motivated to seek out information and improve their diabetes knowledge—a phenomenon known as reverse causation. Therefore, while our data confirm a significant association, they cannot definitively establish whether higher knowledge protects against DKD or whether the presence or perception of DKD drives knowledge acquisition. To clarify the directionality of this relationship, future longitudinal or interventional studies are needed. Prospective cohort studies that measure diabetes knowledge at baseline and then follow patients for the development of DKD would help establish temporal sequence. Alternatively, randomized controlled trials evaluating the effect of structured diabetes education programs on DKD incidence could provide stronger evidence for a causal protective role of knowledge.

Beyond glycemic control (as reflected by elevated HbA1c), our multivariate model identified several other factors independently associated with DKD, offering insights into potential pathophysiological pathways. The strong association between elevated serum creatinine and DKD underscores the central role of declining renal function. In diabetes, chronic hyperglycemia induces glomerular hyperfiltration and subsequent injury, tubular interstitial damage, and podocyte dysfunction, which collectively impair creatinine clearance and elevate its serum levels (22, 23). This process is often intertwined with inflammation and fibrosis, hallmark features of DKD progression (24).

Uric acid is not merely a bystander but may actively contribute to DKD pathogenesis. Recent evidence suggests that soluble uric acid can activate the NOD-like receptor protein 3 (NLRP3) inflammasome within renal intrinsic cells, such as tubular epithelial cells and podocytes, leading to the secretion of pro-inflammatory cytokines (e.g., IL-1β), (25). This sustained inflammatory state promotes renal fibrosis, endothelial dysfunction, and accelerates the decline of glomerular filtration rate. Furthermore, hyperuricemia is closely linked to insulin resistance and endothelial damage, creating a vicious cycle that exacerbates diabetic microvascular complications (26).

Our findings highlight the specific association of elevated triglycerides with DKD. Beyond traditional cardiovascular risk, triglyceride-rich lipoproteins and their remnants can have direct nephrotoxic effects. They may deposit in the glomerular mesangium, promoting mesangial cell proliferation and extracellular matrix expansion. More recently, attention has turned to the role of lipotoxicity and oxidative stress induced by lipid overload in renal cells. Excess free fatty acids and modified lipids can activate inflammatory pathways (e.g., via Toll-like receptors) and induce endoplasmic reticulum stress in podocytes and tubular cells, leading to cellular apoptosis and loss of function (27). This aligns with clinical observations that lipid-lowering strategies, particularly those targeting triglycerides and apolipoproteins, may confer renal benefits beyond cardiovascular protection (28).

## 5 Limitations

This study has several limitations. First, it was a multicenter cross-sectional survey conducted in 15 county-level hospitals in Guangxi. Although rural patients with diabetes were enrolled according to unified diagnostic criteria, the measurement devices for HbA1c, lipid profiles, creatinine, and uric acid varied across hospitals, which may have introduced inter-site variability in test results. Therefore, the generalizability of our findings should be interpreted with caution.

Second, the diagnosis of DKD was based on clinical criteria (albuminuria and/or reduced eGFR) rather than renal biopsy. Although we applied exclusion criteria for known non-diabetic kidney diseases, we cannot completely rule out the possibility of misclassification, as some cases may have had underlying non-diabetic chronic kidney disease.

Third, due to the cross-sectional design, the identified associations cannot be interpreted as causal relationships. The temporal sequence between the associated factors and the onset of DKD remains to be established by longitudinal studies.

Fourth, several important covariates known to influence DKD were not adjusted for in our multivariable model. Data on the duration of hypertension, use of renin-angiotensin system inhibitors (ACEi/ARB), body mass index (BMI), waist circumference, detailed dietary habits, and physical activity levels were not collected. Their absence represents a potential source of residual confounding.

Fifth, we used a uniform HbA1c cut-off of <7% to define glycemic control for the purpose of this study. While this aligns with a standard guideline target and facilitates comparability, it does not reflect the individualized glycemic goals recommended for elderly patients or those with significant comorbidities, potentially leading to an underestimation of adequate control in those subgroups.

Finally, as insightfully suggested during review, a sensitivity analysis excluding patients with macroalbuminuria but preserved eGFR could further test the robustness of our findings. However, in this large-scale multicentric survey focused on clinical prevalence, the granular data required to accurately define this specific subgroup (i.e., individual urinary albumin-to-creatinine ratio values for all participants) were not systematically collected across all sites. Therefore, we were unable to perform this valuable sensitivity analysis. Our results should be interpreted as representative of the overall spectrum of clinically diagnosed DKD in this rural population.

## 6 Conclusion

This study demonstrated a relatively high prevalence of kidney disease among rural patients with diabetes in Guangxi. Higher levels of diabetes-related knowledge were protective against kidney disease, whereas elevated HbA1c, serum creatinine, uric acid, and triglycerides were independent associated factors. In clinical practice, greater emphasis should be placed on health education for rural patients with diabetes to improve their knowledge levels. In addition, closer monitoring and control of blood glucose, renal function, and lipid profiles are essential to reduce the risk of kidney disease and improve patient outcomes.

## Data Availability

The original contributions presented in the study are included in the article/supplementary material. Further inquiries can be directed to the corresponding authors.
